# Advancing the application of systems thinking in health: exploring dual practice and its management in Kampala, Uganda

**DOI:** 10.1186/1478-4505-12-41

**Published:** 2014-08-18

**Authors:** Ligia Paina, Sara Bennett, Freddie Ssengooba, David H Peters

**Affiliations:** 1 Department of International Health, Johns Hopkins Bloomberg School of Public Health, 615 N. Wolfe St., Suite E8541, Baltimore, MD 21205, USA; 2 Department of Health Policy, Planning and Management, School of Public Health, College of Health Sciences, Makerere University, Mulago Hill Rd, P.O. Box 7072, Kampala, Uganda

## Abstract

**Background:**

Many full-time Ugandan government health providers take on additional jobs – a phenomenon called dual practice. We describe the complex patterns that characterize the evolution of dual practice in Uganda, and the local management practices that emerged in response, in five government facilities. An in-depth understanding of dual practice can contribute to policy discussions on improving public sector performance.

**Methods:**

A multiple case study design with embedded units of analysis was supplemented by interviews with policy stakeholders and a review of historical and policy documents. Five facility case studies captured the perspective of doctors, nurses, and health managers through semi-structured in-depth interviews. A causal loop diagram illustrated interactions and feedback between old and new actors, as well as emerging roles and relationships.

**Results:**

The causal loop diagram illustrated how feedback related to dual practice policy developed in Uganda. As opportunities for dual practice grew and the public health system declined over time, government providers increasingly coped through dual practice. Over time, government restrictions to dual practice triggered policy resistance and protest from government providers. Resulting feedback contributed to compromising the supply of government providers and, potentially, of service delivery outcomes. Informal government policies and restrictions replaced the formal restrictions identified in the early phases. In some instances, government health managers, particularly those in hospitals, developed their own practices to cope with dual practice and to maintain public sector performance. Management practices varied according to the health manager’s attitude towards dual practice and personal experience with dual practice. These practices were distinct in hospitals. Hospitals faced challenges managing internal dual practice opportunities, such as those created by externally-funded research projects based within the hospital. Private wings’ inefficiencies and strict fee schedule made them undesirable work locations for providers.

**Conclusions:**

Dual practice prevails because public and private sector incentives, non-financial and financial, are complementary. Local management practices for dual practice have not been previously documented and provide learning opportunities to inform policy discussions. Understanding how dual practice evolves and how it is managed locally is essential for health workforce policy, planning, and performance discussions in Uganda and similar settings.

## Introduction

Dual practice, when health workers employed full time by the government take on additional jobs, is widespread in developing countries, particularly those with growing private sectors. Recent studies found that 29% of physicians in Cote d’Ivoire, 35% of physicians in Vietnam, 42% in Sri Lanka and 41% in Zimbabwe, , and as high as 80% in Indonesia and Bangladesh, held second jobs [1-5]. In some contexts, dual practice can be broader than private for-profit sector service delivery – including both research and NGO work. Researchers and policy-makers in developing countries display increasing interest in how dual practice affects the health system [6,7].

Uganda is one of these countries. The country has a vibrant private health sector. Within this, the private not-for-profit health sector has, for decades, been acting as an extension of the public one, especially after the public health sector was mostly destroyed during the period of civil war and remains underfunded to date. Uganda’s private for-profit sector is large, fragmented, and disorganized, yet very little is known about it. Although there are growing discussions about public-private partnerships in health [8], dual practice seldom features on these agendas and data on this topic is scarce. In Uganda, in 2005, a nationally representative survey of private health facilities found that more than half (54%) of private sector doctors also declared being formally employed in the public sector [9]. While estimates from public facilities do not exist, in general, health providers and policy-makers perceive that almost all government-employed health workers have dual practice. In addition, dual practice has been rising in importance on the policy agenda due to media reports of adverse health service delivery outcomes [10,11], as well as suspected linkages to absenteeism and the wastage linked to it [12,13]. A recent study aiming to establish policy-makers’ research priorities revealed that a principal concern was dual practice that was "*reported to greatly affect the performance of the public sector. The dual* [practice] *of public health workers has implications on quality and management of health care delivery such as indiscipline, time loss and poor work ethics*" [7].

Despite these concerns, data on dual practice in Uganda and elsewhere is scarce. Although many types of health providers are believed to engage in dual practice, the available literature examines dual practice rather narrowly, generally only from the perspective of physicians. Furthermore, existing studies provide few answers to questions related to the policy and management of dual practice, beyond agreement that the effects of dual practice on the organization of the health system and service delivery can be either positive or negative, and that these effects, and related policy responses, are highly dependent on the local context [14-16]. For example, if well managed, dual practice may help prevent doctors from leaving the country by enabling them to supplement salaries without adversely affecting stock of doctors in the country. Conversely, if poorly managed, absenteeism and pilfering may negatively affect public sector standards of care and contribute to inefficiencies. The factors and interactions that drive these effects have not been explored extensively. Presumably, these factors depend on how dual practice has evolved and how it is managed in a particular health system.

Studying the dynamic aspects of dual practice and related interactions both within and outside the boundaries of a health system requires a departure from the linear, theoretical models found in the literature [2,17-20]. A more appropriate model acknowledges the holistic, complex, and adaptive nature of health systems and their broader environment. Complex systems are composed of many interacting components that organize themselves in dynamic ways, are unpredictable in the long-term, and are able to learn from past experiences [21-23]. A research design acknowledging complex systems’ features, as well as potential interactions due to contextual factors, is ideal to guide the exploration of phenomena, such as dual practice, from multiple perspectives. It facilitates the exploration of complex system characteristics, such as feedback, emergence, and self-organization. In this paper, we explore how dual practice evolved in the Ugandan health system and how it is currently managed, in an urban environment – the city of Kampala, with an active private sector. Additionally, using systems approaches, we attempt to reflect on why dual practice persists and the factors underlying its current management. Understanding dual practice holistically in the Ugandan context provides a basis for exploring potential policy options. Gaining an in-depth understanding of the role of dual practice at various levels of the system can help policy-makers and health managers to strengthen management of dual practice and its consequences.

## Methods

### Research design

We use case studies of urban public health facilities to investigate the role of dual practice and the key patterns and interactions that it motivated in the health system. Review of policy documents, as well as qualitative interviews of policy stakeholders were used to supplement a qualitative survey of workers and their managers in the study facilities. In addition, during the data analysis phase, we developed a causal loop diagram to illustrate key factors and related feedback influencing dual practice in the current context. This paper presents only a sub-set of data that were collected as part of a sequential, exploratory mixed methods study.

We purposefully selected five public sector health facilities in Kampala, Uganda, to represent the various levels in the Ugandan government health system: two Health Center III facilities, one Health Center IV facility, and two urban hospitals (see Table 1 for case characteristics). Health Center III facilities have a general outpatient clinic and a maternity ward. Health Center IV is a larger facility than the Health Center III facilities, with the capacity for inpatient services and some emergency operations. Regional referral hospitals have specialized clinics, and are staffed by a variety of cadres, including medical specialists. The national referral hospital also has research and teaching components, in addition to medical service provision. Within these case studies, individual respondents were purposefully selected to ensure that, at each facility, the perspectives of providers (doctors and nurses) and the facility manager, were captured [24]. At each facility, the sample included the health facility manager (in-charge in health centers, directors or department heads at hospitals), as well as a doctor and nurse recommended by the health facility manager. Within the larger hospital, the sampling occurred at the level of the clinical specialty [1], and therefore included multiple manager-level respondents, as well as a nurse and a doctor recommended by each of them. Within the smaller hospital, the sample included the director and two providers recommended by them.

**Table 1:** Summary of selected cases

**Facility type**	**Case A**	**Case B**	**Case C**	**Case D**	**Case E**
Health Center III*	X	X			
Health Center IV			X		
Hospital				X	X
**Location**					
Central		X		X	X
Periphery	X		X		
**Staff composition**					
General practitioners			X	X	X
Specialists				X	X
Nurses	X	X	X	X	X
**Filled positions**	121%	74%	90%	144%	90%

Source: Ministry of Health – Human resources for health audit [25].

*Note: Health Center III units are supposed to be staffed by Clinical Officers and Nurses – although sometimes units do have a Medical officer as well.

Policy stakeholders included purposefully selected individuals from professional councils, relevant government ministries, private not-for-profit medical bureaus, private sector hospital administration, and the local district health office. The main criteria guiding the sampling was the extent to which a stakeholder would know policies on dual practice either at the national level or within their organization, would have a stake in the development of a policy on dual practice, and present a unique perspective on dual practice in the Ugandan context.

### Data collection instruments and field work

A review of policy documents was undertaken before the data collection, and as these documents became available. The main areas of interest were the existence and content of policies, actors, and events that played a role in the evolution of dual practice in Uganda, as far back in time as possible.

The interview guides contained questions about the evolution of dual practice in Uganda, providers’ motivation to engage in dual practice, advantages and disadvantages or challenges linked to dual practice, facility-level policies and management approaches, and potential policy recommendations. Interviews with policy stakeholders focused on policy-related questions, as well as on the evolution of dual practice in the health system. Data collection took place during July–August 2012. The interviews were conducted in English. Interviews were recorded, unless respondents preferred otherwise.

### Data analysis

All of the recordings were transcribed and stored in Atlas.ti v. 7. A preliminary, exploratory coding structure was constructed based on initial readings of the transcripts and on the conceptual framework derived from a systems approach to health markets and theories related to systems thinking and health worker motivation [21-23,26,27]. Multiple rounds of coding focused on refining the scheme [28]. During coding and analysis, memos were developed to capture changes in the coding structure, as well as emerging reflections.

Text query results from Atlas.ti were arranged in matrices for within and cross-case analyses, according to the methods suggested by Miles and Huberman [24]. For each case, matrices were developed by theme (e.g., informal organizational policies), with focus on the embedded units of analysis (e.g., summarizing and contrasting the perspectives of health facility managers, doctors, and nurses). Cross-case theme analyses focused on exploring the differences and similarities between the five cases, specifically by health facility type. The policy stakeholder interviews were analyzed for emerging themes, along the same lines as the case studies. References to the analysis and any quotes are labeled according to respondent type, to maintain anonymity.

### Causal loop diagram development

Although it was not an explicit goal of this study, interviews with policy-makers revealed that the role of dual practice and the government policy on dual practice changed over time, and that examining this progression might be useful for understanding the current policy situation. Based on discussions with policy-makers and case study respondents, as well as available historical accounts, we developed a causal loop diagram (CLD). The CLD illustrates the events, actors, and interactions – or the underlying mental model and system behavior – that fostered the emergence of dual practice policy responses over time in the Ugandan health system, facilitating the visualization of complex system patterns and characteristics, such as policy resistance, feedback, and adaptation [29,30].

The CLD was developed using Vensim PLE Plus [31]. It was challenging to recreate the history of dual practice, particularly in the distant past. An account of the medical profession in East Africa, which included details about the emergence and development of dual practice and the private sector from the perspective of physicians, helped to identify relevant early events from the 1960s and the 1970s [32]. Recent events have been identified from the in-depth interviews conducted for this study and available documents. The CLD was refined through various iterations, to ensure that the relationships, interactions, and direction of feedback were most plausible.

The CLD uses standard notation: "*a positive (+) arrow from variable A to variable B means that A adds to B, or, a change in A causes a change in B in the same direction; a negative (-) arrow from A to B means that A subtracts from B, or, a change in A causes a change in B in the opposite direction*" [33]. Some of the relationships create feedback loops. These loops are reinforcing if the variables influence each other in the same direction. Loops are balancing if they influence each other in different directions. The thickness of the line denotes researcher’s emphasis on a relationship, for illustrative purposes. Dashed arrows highlight key, probable relationships identified through this study. The question mark (?) indicates an unknown relationship. This is not the recommended notation, as it is preferred to make explicit the "*multiple causal pathways connecting the two variables*" [33]. However, data currently do not exist to sufficiently tease out how dual practice affects service delivery. For example, while we know that dual practice can affect systems positively and negatively, whether and how much dual practice contributes to adverse service delivery outcomes is unknown.

### Ethical approvals

Ethical approvals were obtained from the Institutional Review Board of the Johns Hopkins Bloomberg School of Public Health (IRB No. 4371), the Makerere University College of Health Sciences – School of Public Health Higher Degrees, Research, and Ethics Committee (IRB No. 11353), the Mulago Research Ethics Committee (Protocol no. 249), and the Uganda National Council for Science and Technology (Ref. No. SS 2883).

## Results

Twenty-three interviews with doctors, nurses, and health managers from various types of facilities, as well as 13 policy-stakeholder interviews were conducted. None of the respondents approached for an interview declined to speak to us, although a few preferred that our interview not be recorded. Respondent characteristics are displayed in Table 2. About half of our health facility respondents reported having dual practice at the time of the interview, or having been previously been involved in private sector work.

**Table 2:** Interview respondent characteristics

**Facility-based respondents**							
		**Case A**	**Case B**	**Case C**	**Case D**^**1**^	**Case E**	**Nr. (%)**
**Gender**	Male	0	1	1	2	4	8 (35%)
	Female	3	2	2	1	7	15 (65%)
**Yrs. in service**	<10	1	1	1	0	0	3 (13%)
	10–19	0	1	1	0	5	7 (30%)
	20–29	2	0	1	1	2	6 (26%)
	30+	0	1	0	2	3	6 (26%)
**Profession**	Nurse	2	2	2	1	2	9 (39%)
	General practitioner	0	1	1	0	0	2 (9%)
	Clinical officer	1	0	0	0	0	1 (4%)
	Specialist	0	0	0	2	9	11 (48%)
**Dual practice**	Yes						10 (43%)
**TOTAL**							**23**
**Policy stakeholders**							
**Gender**	Male		12 (92%)				
	Female		1 (8%)				
**Sector**	Public/government		5 (38%)				
	Professional associations		4 (31%)				
	Private for-profit		3 (23%)				
	Private not-for-profit		1 (8%)				
**TOTAL**			**13**				

^1^Years in service not available for one of the respondents at this facility.

The CLDs that follow display the factors associated with the presence of dual practice in the system and the emergence of current management practices and policies. They illustrate three phases to describe the emergence of dual practice in Uganda: pre-independence through the 1960s, 1970s through the 1980s, and the 1990’s through the present. Table 3 complements the CLDs by illustrating a timeline of critical events that affected the policy and management of dual practice.

**Table 3:** A timeline of critical events and government policy on dual practice

**Year**	**Event**	**Dual practice policy**	**Consequences**
**Pre-**	Nr. of African health professionals growing	Weak formal govt. restrictions: dual practice allowed after govt. hours	None
**1962**	Ugandan independence		
**Post-1962**	Govt. suspicions about private sector growing	Strong formal govt. restrictions: dual practice not allowed	No immediate effects
**…**			
	Transition to military rule and civil war		
**1972**	Asian doctors expulsed		After 1970’s events, restrictions to dual practice contributed to resignations from government services and provider migration – therefore reducing the number of govt. providers
**…**	Ugandan doctors take over private practices		
**1974**	Government shuts down private practices		
**…**	Provider protest advocacy to allow dual practice		
**Late 1970’s**	Broadly, international sanctions on military government led to economic collapse and decline in government salaries relative to cost of living	Weak formal govt. restrictions: dual practice allowed after govt. hours	Dual practice is a coping mechanism for providers remaining in Uganda
	Government changes policy on dual practice as incentive for govt. providers		
**1980’s**	Govt. suspicions about dual practice and private sector strengthen	Weak, formal govt. restrictions: dual practice not allowed	
**1990’s**			
**2000’s**	Rapid private sector growth, especially after system recovered from civil war, creates increasing nr. of dual practice opportunities	No formal govt. restrictions	
**…**			
		Informal govt. restrictions on dual practice, with weak influence	
**2005–2007**	MOH tests ban on dual practice in few hospitals		Providers threaten to resign
**2009–2010**	Office of President establishes Medicines and Health Service Delivery Monitoring Unit		Dual practice important coping mechanism
	Increasing nr. of policy discussions around dual practice, absenteeism, ghost workers		Providers threaten to resign in response to discussions of ban
	Increasing concerns about the contribution of dual practice to decreases in quality and access to care in both public and private sectors		

The remainder of this section first describes, in each phase, the feedback and interactions that emerged in relation to government policies on dual practice, as well as, more broadly, the development of a mixed health system in Uganda. It concludes by describing how dual practice is currently managed in the government facilities included in this study.

### Phase 1: Dual practice policy before Uganda’s independence and through the 1960s

**Figure 1:**
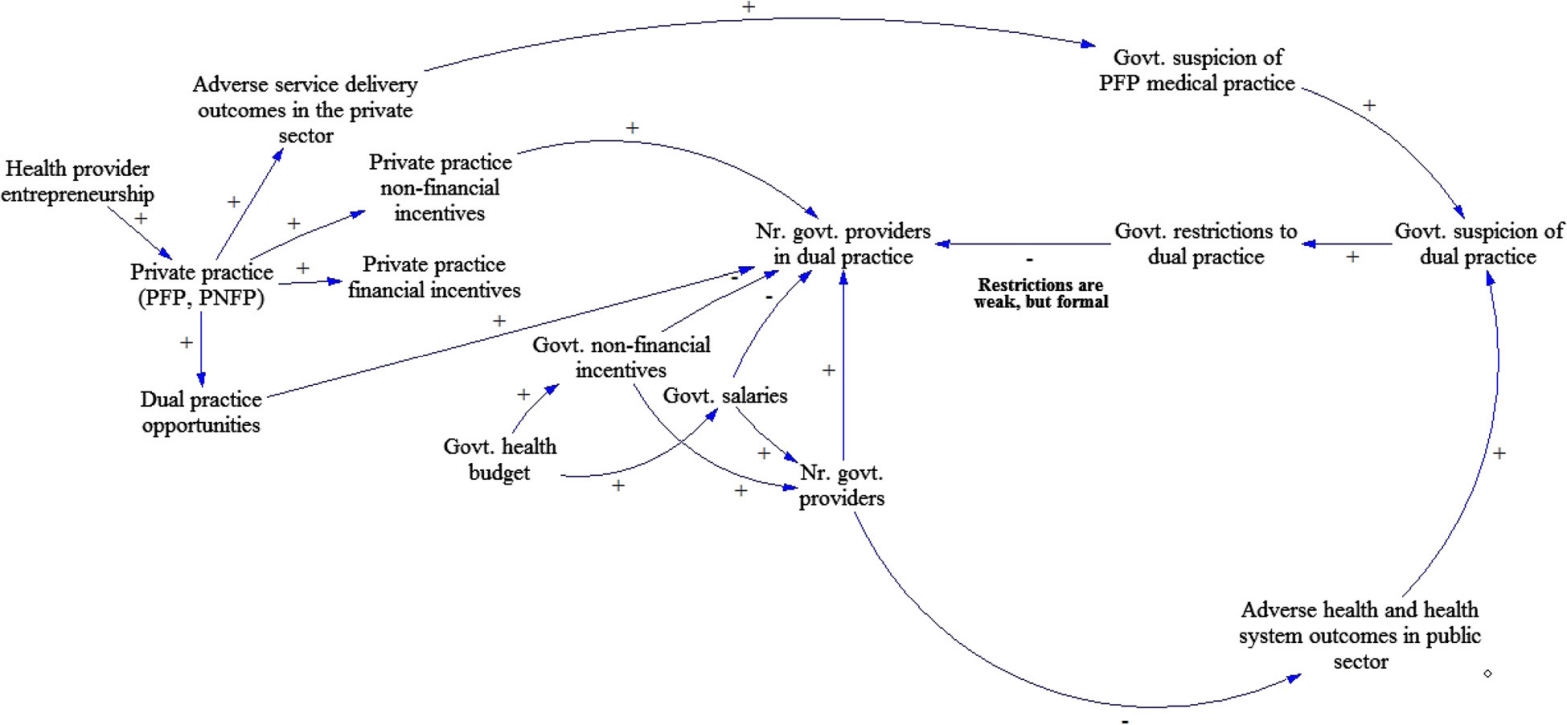
**Causal loop diagram illustrating factors influencing dual practice from pre-independence through the 1960’s.** The causal loop illustrates the first period of interest: a simple system with little demand for dual practice. It is important to highlight that no feedback loops were identified in this phase.

Figure 1 illustrates a relatively simple system, showing no feedback or unintended consequences, where a nascent private sector does not initially provide sufficient incentives for providers to engage in dual practice.

During this time, government restrictions on dual practice are formal – written and enforced: dual practice is allowed only after government hours. Some dual practice opportunities exist, however, demand is low due to high satisfaction with the government benefits. Few government providers chose to engage in dual practice, generally seeking the autonomy provided by private practice. No evidence was found that dual practice raised concerns about adverse health service delivery outcomes. Even as restrictions on dual practice became stronger after Uganda’s independence, the Ugandan government was able to provide government health workers with sufficient financial and non-financial incentives (e.g., satisfactory wages and the prestige of working in a government institution, respectively). One of the policy-stakeholder respondents confirms the general sentiment in this period [32]:

"*The assumption was, that what the government pays can cater for what you require in real life.* […] *in the 60’s, a medical officer, medical assistant, the nurse, was capable of catering for everything they required, the basics of life* [with the government salary alone]*. And they were held with high esteem, they were very ethical. I mean a medical officer would walk with his head high.*" *–* Ministry of Health policy stakeholder

### Phase 2: Dual practice policy in the 1970s and 1980s

**Figure 2:**
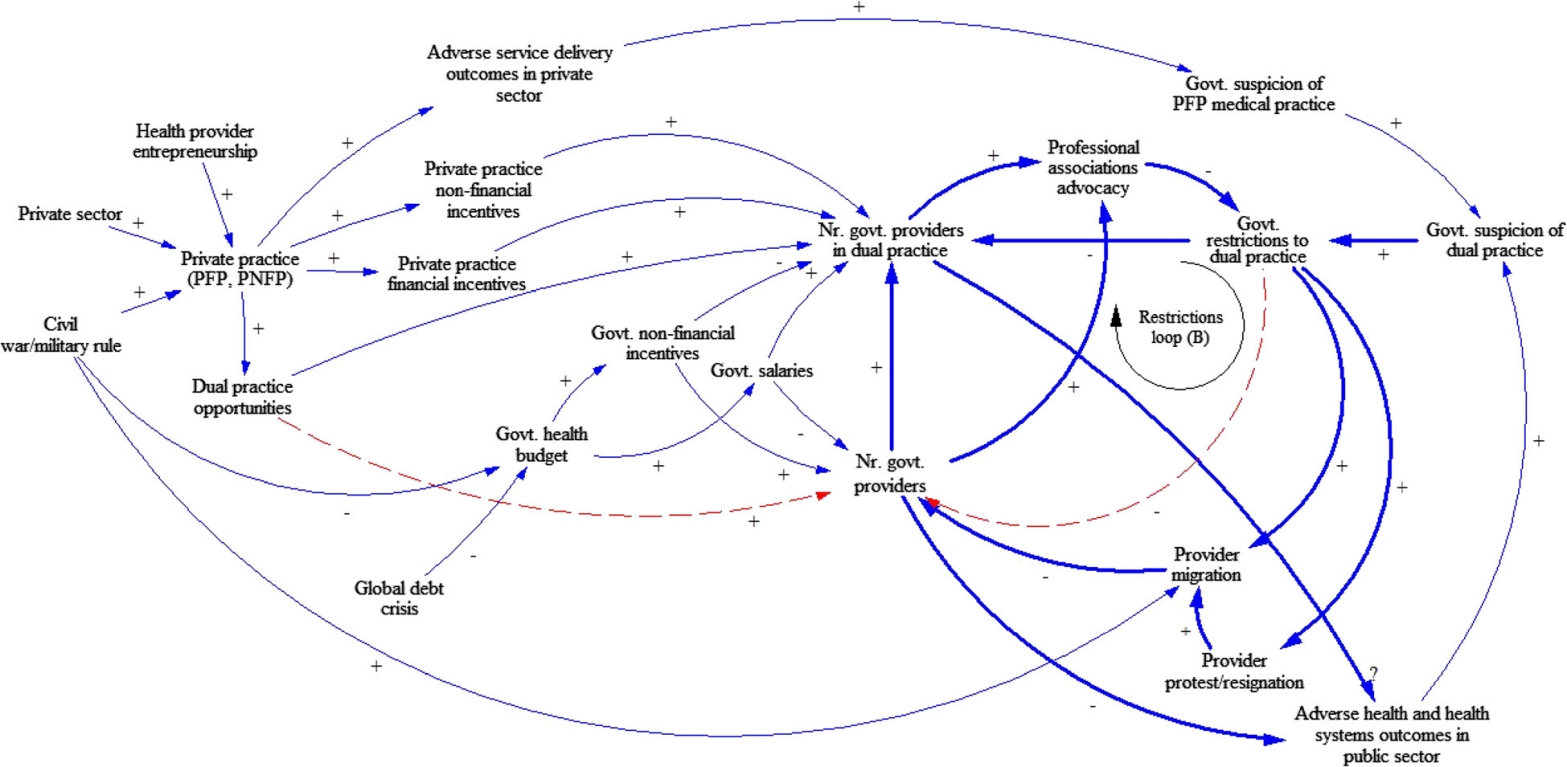
**Causal loop diagram illustrating factors influencing dual practice during the 1970’s and 1980’s.** The causal loop illustrates the second period of interest: the health system is challenged by broader contextual events – such as the civil war and the global debt crisis. As demand for dual practice grows, so do opportunities for government providers. The government becomes increasingly suspicious of potential adverse effects and, at first, imposes a ban on dual practice. A balancing loop first results in unintended negative consequences (See Figure 3 for further details).

Figure 2, illustrates a second phase, during which the Ugandan system undergoes instability of military rule and, eventually, civil war.

**Figure 3:**
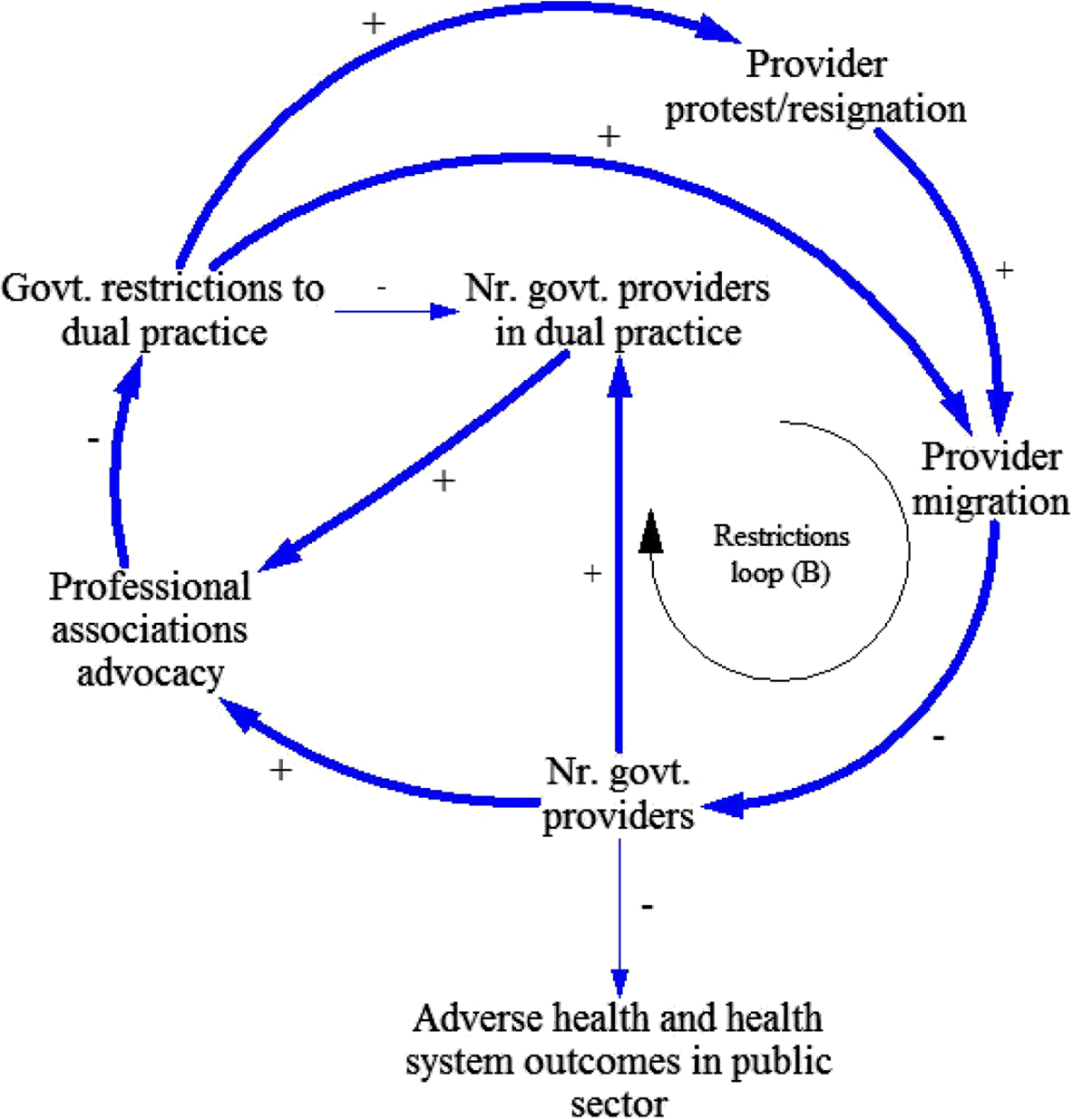
**Focusing on the restrictions loop.** Starting with the 1970’s, strong restrictions to dual practice trigger unintended consequences through a balancing feedback loop – a decrease in the number of government providers. Subsequently, successful advocacy efforts to ease restrictions eventually dampen their effects on the broader health workforce, although restrictions remain in place they are acceptable to the provider population. This figure re-draws the CLD diagram to better illustrate the factors influencing these unintended consequences.

During this period, instability affects the health workforce in multiple ways: through reduced infrastructure and government budgets, as well as through persecution of health providers for political reasons. These country-level hardships are intensified by the broader global recession. The multiple crises cripple the government health system, and mark the beginning of several decades of low government salaries. While the job security and prestige related to government service are still important, the government financial incentives are no longer sufficient for providers who remain in the system. Many government providers resign at this time, or leave the country. Increasingly, government providers who remain in the system seek additional income through dual practice. The same policy stakeholder explained:

"[With] *the economic downturn of the 70s, then the wars that have been associated with* [Amin and Obote’s] *regime, the salary did not have any meaning anymore.* […] *The global economy has changed, impacting everyone,* […] *the country, with all the hardships it’s had – the economy has not been able to cope with the many social needs. That’s why salaries across all public servants have remained very low and therefore public servants have to look for alternative survival mechanisms.*" – Ministry of Health policy stakeholderAs the public sector increasingly suffers and government providers "look for alternative survival mechanisms", this period leads to the first large-scale development of the private sector, after Asians (including doctors) were expelled from Uganda for political reasons, many of the Ugandan government doctors who remained in the country re-opened the former Asian private practices and many of them were perceived to have dual practice. At this time, the increasing concerns about the quality of services provided in private for-profit medical practices (while not proven to be linked to adverse health and health system outcomes), contributes to suspicion around dual practice, specifically related to potential damage to the quality of services in the public sector and pilfering of government medical supplies. Consequently, the government begins imposing strong, formal restrictions on dual practice, a strict ban on dual practice and, at one point in 1972, closes all private clinics. As shown in Figure 3, these strong, formal restrictions to dual practice trigger provider protests and resignation, and contribute to provider migration, both of which compromise the supply of government health providers. International sanctions on the military government and a declining economy made salaries of civil savants unattractive. Increasing protests and advocacy from professional associations eventually lead to the government relaxing restrictions. Weaker restrictions, which allowed dual practice after government hours, reduced the threat to government providers and diminished the undesirable feedback.

During the 1980s, the global debt crisis and the subsequent structural adjustment program fuelled the development of the private sector, while, at the same time, constraining government budgets [34]. In this context, the financial benefits of working in private practice significantly exceeded those of the public sector, and motivated government providers to engage in dual practice. Government restrictions remain formal, but weak, at this time. Based on the available information, we propose that during this eventful and tumultuous period, dual practice and the incentives related to practicing in the private sector complement incentives for government service. Moreover, restrictions on dual practice without any further measures to address the government health system contribute to a decrease in the number of government providers. The dashed lines in Figure 2 highlight these proposed influences.

### Phase 3: Dual practice policy from the 1990s to the present

**Figure 4:**
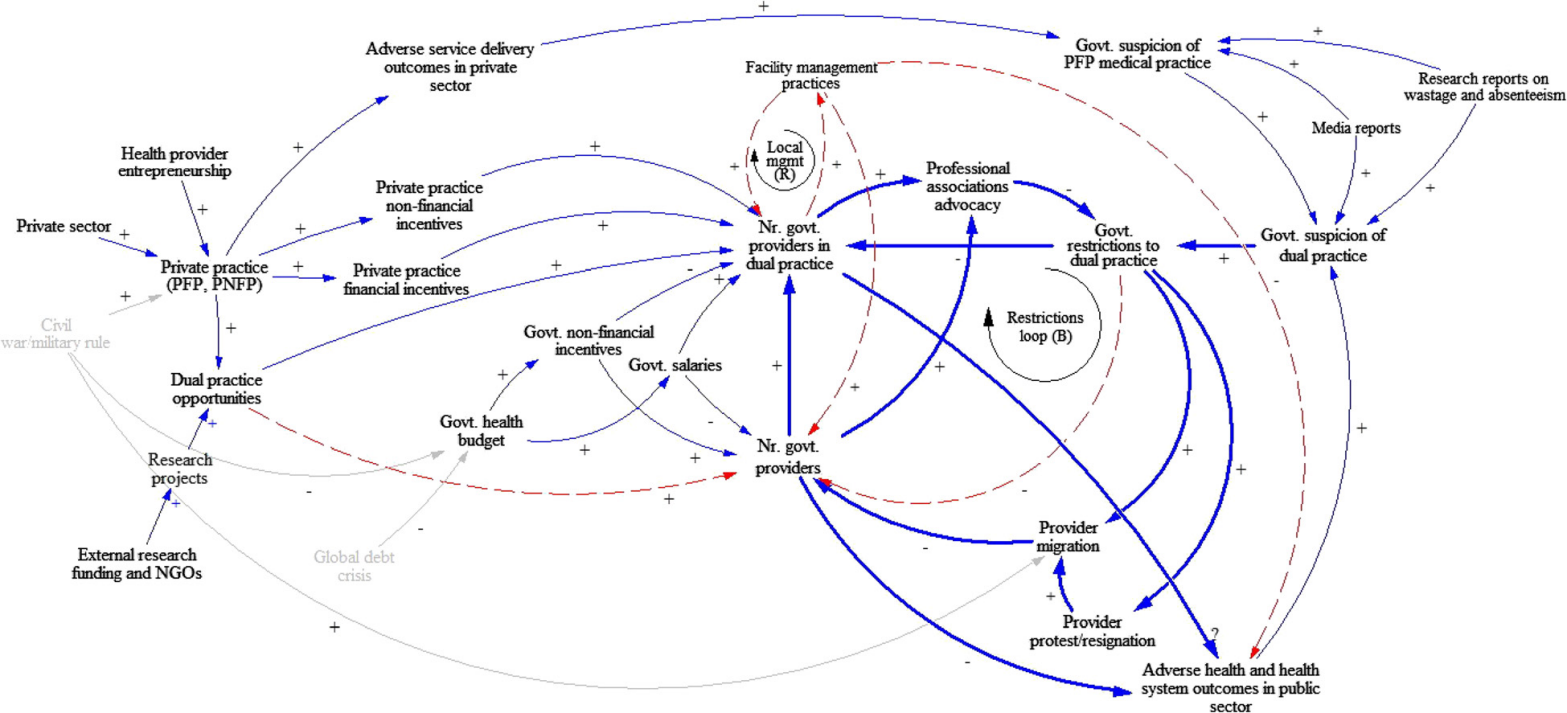
**Causal loop diagram illustrating factors influencing dual practice during the 1990’s to the present.** Dual practice opportunities grow exponentially, as it becomes more attractive to government providers working in an underfunded and over-burdened public system. A formal, written government policy does not exist. Local facility-level coping mechanisms emerge to mitigate negative consequences of dual practice on the health system. Periodic threats for increasing restrictions re-activate the feedback loop presented in Figure 3.

Figure 4 illustrates the changes in the system from the 1990s to the present: the private sector grows significantly as Uganda recovers from civil war and privatization is encouraged through the structural adjustment program [34] and well-financed vertical health projects and clinical research initiatives.

Dual practice opportunities grow quickly in a context of rapid private sector growth, as well as of increasing donor-funded research and NGO projects, generally housed within public facilities. Due to an ever constrained budget and growing population demand, the government health system cannot offer providers an alternative to dual practice. Private practice during this period promises significant financial incentives, particularly in contrast to low government salaries, but lacks the job security and prestige that are still associated with government practice. The increasing population demand, as well as the significant earnings possible through private practice, make dual practice a frequent coping mechanism for government providers. In the absence of formal rules to manage dual practice, health facility managers develop their own formal and informal practices for mitigating detrimental effects of dual practice, such as absenteeism, while retaining the government health workforce, despite low salaries and poor infrastructure.

The absence of a formal policy on dual practice was confirmed by interview participants and also by our review of Ministry of Health and Ministry of Public Service policy documents. As our respondents illustrate below, current government restrictions are informal – unwritten, not enforced, and based on expectations of provider behavior in the public sector.

"*I don’t think there is a clear policy saying that there is no dual practice* […] *we are expected NOT to do it* […]. [Health workers] *know what’s supposed to be the normal, but are kind of forced to do it, as I’ve said, to improve a bit on their earnings.* […] *We don’t come out to fight it. I can’t tell someone please don’t go the other end, because there’s a reason that is pulling this person to go, and I have no control over that. All I can do is to make sure enforcing that this person is here with me at the right time, for 7 or 8 hours. So we can’t influence what happens beyond that* […] *I cannot influence the earnings.* […] *The person has the needs, and I can’t satisfy the needs in any other way* […] *I can’t provide alternatives.*" – Government official 1

"*A lot of policies are implemented while they are just known by the policy-makers but they are not written down. So, we know about dual practice and the policy is that* […] *it should be left as it is. That people can be allowed to do dual practice.* […] *It is not written. It’s not written at all, but they should not take too much of public time to do it.* […] *Unfortunately, there is no mechanism to enforce how much public time people are going to take because* […] *a lot of things that have gone wrong, including this dual practice, have gone wrong because of poor regulatory systems.*" – Government official 2

Interviews with policy stakeholders revealed that the government initiated periodic attempts to formalize government restrictions on dual practice, motivated by suspicion around dual practice due to media coverage of adverse health outcomes and poor public sector performance. Additionally, restriction attempts are triggered in the context of budget discussions, media reports about ghost workers, and increased concerns about quality of care in both public and private sectors as indicated by adverse health service delivery outcomes linked to absenteeism, pilfering of drugs, and patient deaths in the private sector from suspected malpractice.Escalating policy discussions around formalizing restrictions on dual practice are often met with provider protests, triggering the feedback displayed in Figure 3 and the government goes back to "keeping quiet", in this case meaning informal restrictions. A couple of the policy stakeholders provides examples of such events, which also illustrate that the government increasingly recognizes the role of dual practice in the system, particularly in the absence of changing government pay.

**~2005–2007***:* "*The* [high level official] *gave a directive that it should stop.* […] *He said: ‘Officer, we are going to work out the methodology of implementing it* […] *But we shall not do it broadly across the country, we shall test it in some hospitals.’ So we came* [to one of the hospitals]*, and communicated what the* [high level official] *had done, and said, these people* [at this hospital] *said: ‘We hear you loud and clear, but let’s agree if I cannot take that prescription, am I free to leave the government job? So that I can go to the other side* [meaning private practice]*?* […] *we either stay or go? Is that what you’re trying to communicate to us?’ We said: ‘Yes’. Within two days* […] *the* [hospital] *director came rushing to the headquarters to say: ‘Guys, stop talking about dual practice because everybody is winding up to go.’ So, the* [government official] *went back and told the* [high level official]*: ‘We tried to test it in* [a hospital] *and all the consultants are not bothered - they want to leave* [this hospital]*.’ - and dual practice has gone on*." – Ministry of Health policy stakeholder 1

In response to the cycles of uncertainty related to informal government restrictions to dual practice, as well as to coping with potential negative consequences of dual practice on public sector performance, we found that local, facility-level and department-level management practices can develop in government facilities. Facility management practices arise in response to increasing number of government providers with dual practice and aim to reduce any adverse service delivery outcomes in the public sector. As long as dual practice remains an incentive for providers to remain in government service (and sufficient resources to incentivize providers otherwise do not exist), these facility management practices could potentially weaken any policy restrictions to dual practice and any related negative effects on the government workforce.

### Local management practices for dual practice

Data from the case studies revealed that, in the absence of a formal, written policy on dual practice, health managers develop their own approaches to coping with and managing dual practice on a daily basis. Table 4 summarizes the approaches identified through this study. These facility-level management practices encourage the presence and performance of their staff during government hours although these codes of practice are generally unwritten. For example, no respondents described dual practice being addressed directly during regular staff meetings. Instead, respondents described informal, one-on-one consultations: health managers intervening with providers in private, often in response to an issue related to provider performance.

**Table 4:** Facility-level management practices for dual practice, by case

	**Facility-level management practices**	**Attitude for dual practice**	**1-on-1 consultations**	**Discussion in staff meetings**	**Incentives/support supervision**	**Effect on the supply of government providers**
**Case A**	Dual practice allowed after government duties completed	Negative	Yes	No	No	Associated misunderstandings potentially create feedback that decreases the supply of government providers. Providers interviewed had a different interpretation of the in-charge’s version of "completeness," and reported leaving government work early. The misunderstandings associated with this approach were perceived to result in absenteeism
**Case B**	Motivate providers to perform at their public sector job (e.g., supportive supervision; tea, purchased in health manager’s personal funds); non-interference with health workers lives outside government duties	Cautious	Yes	No	Yes	Potentially promotes desirable feedback, by creating conditions to improve public sector performance and retain government providers
**Case C**	Discourage dual practice; emphasize priority for government duties and high public sector performance	Negative	Yes	No	No	Potentially promotes undesirable feedback by reducing the number of government providers; alternatively threats of disciplinary action could support improved performance in public sector
**Case D**	Priority for government duties; non-interference with time outside government duties	Positive	Yes	No	No	Potentially does not affect government supply of doctors, but creates tensions among staff
						Although the Case D – the smaller hospital’s leadership had a positive attitude towards dual practice, they did not report a specific management strategy, except non-interference. Doctors reported to cope with dual practice through individual negotiations among their colleagues; however, this was not without pitfalls, as nurses were perceived to compensate for the absence of doctors. Furthermore, doctors appeared to have difficulty responding to emergencies, given that they juggled two or sometimes more places of work
**Case E**	**Formal policies**	Mixed, depends	Yes	No	Yes, in the context of flexible scheduling; N/A for other policies and practices	Potentially effective at reducing the number of nurses working two full time jobs. According to respondents, also improved attendance among nurses. Probably no effect on those with part-time dual practice
	Policy preventing nurses to sign up for only night duties (which typically means they have a full-time day job)					
	A memorandum of understanding with externally funded research projects, to stop the active recruitment of government staff to fill full-time positions on projects					Effective at reducing active recruitment by research and NGO projects, therefore reducing internal dual practice opportunities. According to respondents, also improved attendance among nurses
	Private wing					Ineffective – mild effect on government providers, but has potential if more efficient.
	**Informal policies**					Sustains retention among government providers, particularly specialists. Flexible scheduling creates friction among non-physicians
	Flexible scheduling					

In one case, a health manager fostered a culture of flexible scheduling, i.e., all senior doctors get one day or certain afternoons to dedicate to their other activities, whether research or dual practice, in exchange for reporting to duty on other days. According to the unit’s manager:

"*We tried to create a bit of flexibility and say, ok, all of us must be on station in the morning, and let’s take turns to cover the evening. And may be trying to bring the evening time a bit forward to, to allow people to earn some extra earning.* […] *When I see the outputs, then I don’t complain. Yes, and sometimes they come and start early, before 8 o’clock and if someone is here by 7 and even comes back on the weekend to clear if there is any backlog, I think, really, I can only say thank you because I can’t pay them more than they earn.*" – Case E, Health manager

This particular arrangement was not only facilitated by the fact that the unit manager was understanding of the reasons why providers would engage in dual practice and had an output-oriented supervision style, but also by the fact that the majority of doctors working in this unit worked in the same private health facility, which was close to their government location.

Within the larger facilities, formal policies included, for example, having a private wing, where doctors and nurses could see and get paid for private patients under the auspices of the government facility, or limiting nurses’ night duties so as to deter them from taking up full-time dual practice during the day.

Most of the health managers interviewed had a generally favorable attitude towards dual practice, not discouraging it within their facility. Their attitude stemmed from their own personal experiences where, in the past, they also had no choice but to take on additional jobs to compensate for government sector shortcomings. It also stemmed from broader frustration at not being able to enforce attendance policies and not having the necessary tools to adequately monitor health workers (an exception was the health manager for Case C, who expressed high confidence in public accountability). The principal tool available to managers for holding health workers accountable were attendance registers, which could be easily falsified. In this context, dual practice was generally tolerated within government facilities. Health managers emphasized the need to prioritize the completion of government duties and, to the extent possible, tried to introduce incentives for improved performance in the public sector.

"*I don’t stop anybody from doing that. What* […] *I tell them is that: priority is a core job and your core job is the public service. Once you do my work well, then I don’t mind about what you do next.*" *– Case D, Nurse/Health Manager*

These management approaches, generally lenient with respect to dual practice, seemed to mitigate providers’ exit from the government health workforce. Additionally, they also seemed to tackle broader issues of provider performance, such as absenteeism. In Case B, providers reported being able to manage their two jobs without conflict. One of the providers reported seeing dual practice as a privilege: "*if you want to reward yourself by doing an extra job, you have to make sure we* [in the government sector] *are covered*" (Case B, doctor). Nevertheless, because of the broader health system issues, where managers lack tools to properly enforce policies in general, approaches to manage dual practice also had shortfalls. For example, in Case C, the in-charge reported that providers who were found with multiple jobs (often caught in the private facilities), were asked to quit them in favor of government service. While this manager reported confidence in this approach, the other respondents from the facility reported that almost everyone in the facility engaged in dual practice, but this was not discussed with the manager. Some of these approaches also created tensions among staff. The flexible scheduling mentioned earlier was not available to non-physicians and therefore friction arose from time to time among work teams. The private wing is one of the dual practice policy interventions listed in the literature [15], however, in the study context, it was perceived to be inefficient, and the infrastructure only marginally better than the rest of the facility.

## Discussion

This paper is one of the few contributing empirical evidence on dual practice policy and management practices in Uganda and low- and middle-income countries, more broadly. It illustrates how dual practice policies changed over time in the Ugandan system and how this phenomenon is currently managed within a sample of government facilities. It also attempts to use the existing data to reflect on and to explain why dual practice persists and the current approaches that have developed in the study context.

Due to a series of health and non-health sector events, feedback, and learning, dual practice has become an informal, yet integral component of a government health workers’ incentive package. This package has also evolved over time to one where job security and prestige remain important, but no longer sufficient due to some of the lowest salaries in the region, poor system infrastructure, and increasing patient loads. In a situation where the government cannot offer financial or non-financial alternatives to substitute dual practice (i.e., improve the incentives for sole public practice), the official policy for dual practice shifted from formal restrictions to one based on informal expectations. Any attempts to formalize restrictions is met with unintended consequences due to policy resistance and emerging feedback, threatening the stability of government health workforce more broadly.

Our data confirmed the existence of self-organization through local, facility-level management practice, which allow health managers and providers to cope with working in both the public and private sectors. The purpose of these practices was not necessarily to curb dual practice, but to maintain performance of the public sector by ensuring the presence of providers and, at the same time, to achieve an optimum balance between government workers’ public and private activities and needs. Some of these management practices were easy to identify and describe, e.g., the ones guided by a health manager, as in the example of the hospital department. Other management practices, based on individual negotiations, presumably depended on internal provider networks, whose development, and also decline, could not be captured through our study methods. Most frequently, health managers found opportunities to intervene as common symptoms of dual practice that threaten public sector performance, such as absenteeism, triggered concerns. These management practices could potentially minimize destabilizing effects occasionally arising from the policy feedback and resistance.

Our exploration revealed two issues that are relevant beyond the issue of dual practice policy and management and perhaps also beyond the Ugandan context. First of all, public sector performance management emerged as an area with significant shortfalls. In the absence of tools and support for rewarding good performance and punishing poor performance, the tacit, indirect approach to managing dual practice does not sufficiently empower health managers to supervise and enforce boundaries for government employees, who must fulfill their duties in both the public and private sectors. Also, because the nature of dual practice differs among nurses, general practitioners, and specialists, cadre-specific management approaches and tools might be appropriate.

Second, the nature of the Ugandan health system, and that of many sub-Saharan African countries, is very different than it was immediately after independence. Initially designed around the public sector, the private sector and particularly the private for-profit components, have been treated with suspicion and not integrated within a broader vision for the health system. Presently, the pluralist health systems that dominate low- and middle-income countries cannot be ignored. As a majority of the population, including the poor, relies on the private for-profit sector, increasing government stewardship is necessary to maintain the highest standards of service delivery [35]. In this context, providers engaged in dual practice could serve as a channel for reaching the private for-profit sector and the synergies between government practice and private for-profit practice must be recognized.

How dual practice actually affects the health system and service delivery remains one of the key unknowns. While the literature and study respondents acknowledged both positive and negative effects of dual practice, in most low- and middle-income contexts, Uganda included, actual effects on the health system are unknown. Furthermore, issues such as shortfalls in quality of care, absenteeism, and efficiency gaps in public spending have broader root causes and can only partly be attributed to dual practice. A better understanding of the dual practice effects on providers, health facilities, and the broad health system would help governments to better calibrate their policy approach and to explore options for reaching a better balance between public and private sector spheres in health care.

### Strengths and limitations

This study represents one of the few exploring dual practice holistically, from multiple perspectives (doctors, nurses, managers, policy stakeholders), and by applying systems thinking tools, such as the CLD. Only a few examples of CLDs exist in health research [36-39]. The researchers established credibility and confirmability of the findings by triangulating the data from the interviews across multiple types of providers, and, where possible, through the document and policy review.

The conclusions are constrained by several limitations. Much of the early history of dual practice in Uganda relies on a single source and it was not possible to verify the events or written government documents we mention. Because the case studies were based in a large urban center, generalizations to rural Uganda, where the opportunities for private practice are substantially different, are not possible. We could not explore dual practice in private not-for-profit or for-profit facilities or include additional cadres believed to engage in dual practice (e.g., clinical officers). The large hospital was much more complex than the other cases included in our study, and perhaps deserved to be studied in greater depth. Although the information presented in the CLD was triangulated across all available data sources, it could not be validated with study respondents as it was developed after the data collection ended.

Future research into how dual practice is managed by public facilities and how private for-profit facilities incentivize and contract with their providers, would be helpful. More in-depth studies looking at dual practice from the perspective of other cadres, such as clinical officers, or of rural practitioners could provide additional insights into this phenomenon. The facility-level management mechanisms described here could inspire formal policies aimed at minimizing the negative consequences of dual practice, while helping to seize opportunities for public-private sector synergies. The effects of dual practice on service delivery outcomes, such as quality of services and access to care, have not been established in the literature, although there is consensus that dual practice likely contributes both positively and negatively. Validating the CLD and translating it into a system dynamics model could be relevant in policy discussions as a platform for testing various policy scenarios and anticipating unintended feedback in the system.

### Policy relevance

The unintended feedback revealed through the CLD, at times detrimental to the public health sector, confirms the recommendations of previous studies, which proposed that a ban on dual practice would not be practical or effective [14-16,40]. Periodic threats of banning dual practice also risk destabilizing the public health sector in places like Uganda, primarily by reducing the supply of government health workers. The private for-profit sector allows government providers the additional financial resources that the Ugandan government is currently not able to supply. In a relationship of mutual dependency, government providers in dual practice allow for the growth of the private for-profit sector in the context of limited health workforce and increasing population demand.

In the short term, the Ugandan government should consider the promotion of policies that are flexible to local adaptions to promote access and quality of services in the public sector, while at the same time allowing sufficient income for government providers. Informal, local adaptations to managing dual practice exist in Uganda and provide a natural experiment for various dual practice policies. In the long term, the Ugandan government should consider broader improvements to public sector management and increasing the resources available to the health sector, as well as increasing synergies with the private sector.

Reforms currently under discussion in Uganda include health insurance and performance-based contracts, both would change how providers are paid. Such reforms could potentially provide an entry point for strengthening public sector management in general, and therefore provide health facility managers the tools they are currently lacking to manage dual practice. As dual practice is unlikely to disappear in the short term, its existence and role in the health sector cannot be ignored during the design and implementation of major health reforms in Uganda and other countries where dual practice exists. Potential unintended effects (feedback) should be anticipated based on past events related to dual practice and dealt with accordingly.

## Competing interests

The authors declare that they have no competing interests.

## Authors’ contributions

This study was part of LP’s doctoral dissertation. DP, SB, and FS were part of the advising and thesis committees, making important contributions to all phases of design, implementation, and analysis. LP prepared the first draft of the manuscript. DP, SB, and FS contributed to revisions and finalizing the manuscript. All authors read and approved the final manuscript.
